# Carnitine Deficiency after Long-Term Continuous Renal Replacement Therapy

**DOI:** 10.1155/2022/4142539

**Published:** 2022-08-17

**Authors:** Caroline Van de Wyngaert, Joseph P. Dewulf, Christine Collienne, Pierre-François Laterre, Philippe Hantson

**Affiliations:** ^1^Department of Intensive Care, Cliniques St-Luc, Université Catholique de Louvain, 1200 Brussels, Belgium; ^2^Department of Clinical Chemistry, Cliniques St-Luc, Université Catholique de Louvain, 1200 Brussels, Belgium; ^3^Louvain Centre for Toxicology and Applied Pharmacology, Université Catholique de Louvain, 1200 Brussels, Belgium

## Abstract

A 60-year-old man was admitted in the intensive care unit (ICU) for a rapidly progressive respiratory failure due to SARS-CoV-2 infection. He developed numerous complications including acute kidney injury (AKI) requiring prolonged continuous renal replacement therapy (CRRT). Enteral feeding was initiated on day 8. Despite nutritional management, there was a remarkable amyotrophy and weight loss. On day 85 in the ICU, the patient became progressively unresponsive. An extensive metabolic workup was performed, and blood results showed hyperammoniemia and hypertriglyceridemia. Plasma free carnitine level was low, as was also copper. After carnitine supplementation, the neurological condition rapidly improved, and metabolic perturbations regressed. Prolonged CRRT may be complicated by clinically significant deficiency in micronutrients and trace elements.

## 1. Introduction

During the recent COVID-19 pandemic, the incidence of acute kidney injury was particularly high, and numerous patients required prolonged renal replacement therapy (RRT) [1]. Among the unusual complications of continuous renal replacement therapy (CRRT), deficiency in some micronutrients may be of concern [2, 3].

We report a case of secondary carnitine deficiency following CRRT of a particularly long duration.

## 2. Case Description

A 60-year-old man (84 kg body weight, 25.1 kg/m^2^ body mass index) with no medical past history was admitted in the intensive care unit (ICU) on November 2021, 9 for progressive respiratory failure due to SARS-CoV-2 infection. He rapidly required orotracheal intubation for mechanical ventilation. The clinical course was characterized by early complications including atrial fibrillation and arterial thrombosis in the right forearm. Enteral nutrition was initiated from day 8 via a nasogastric tube. It consisted of Fresubin® HP Energy (1.5 kcal/mL) (Fresenius Kabi, Belgium), containing: lipids 5.8 g/100 mL, carbohydrates 16.2 g/100 mL, proteins 7.5 g/100 mL, zinc 12 mg/100 mL, copper 130 *μ*g/100 mL, and selenium 6.7 *μ*g/100 mL. More specifically, this diet was containing 0.66 g/100 mL of lysine and 0.23 g/100 mL of methionine as potential precursors of carnitine. The mean daily caloric intake was 1472 kcal over the first two months (excluding glucose intake from basal dextrose infusion and lipid intake related to sedation with propofol). The patient was also regularly supplemented in thiamine, vitamin K, hydroxocobalamin, iron, and folate. He never received any medication (valproate, phenytoin) known to lower carnitine levels. There was also no recent infection by urease-producing bacteria.

After the development of oliguria and acute renal failure, continuous venovenous hemofiltration (CVVH) was started from day 41. Regional citrate anticoagulation was administered before the filter (AN-69®; 0.6 m^2^), and the predilution and postfilter fluid flow with Prismocal® was 1000 mL/h each; hemofiltration rate was adapted to maintain a negative fluid balance.

**Figure 1:**
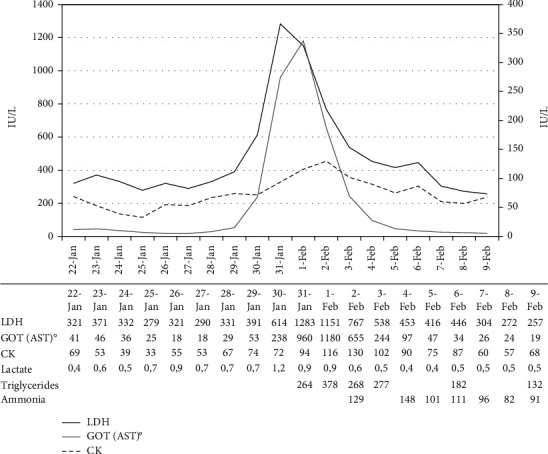
Laboratory investigations. Normal range: LDH < 250 IU/L, AST 19-48 IU/L, CK 20-200 UI/L, lactate 0.5-2.2 mmol/L, triglycerides < 150 mg/dL, ammonia < 90 *μ*g/dL.

Physical examination revealed an impressive weight (-14 kg) and muscle loss. The patient was found poorly responsive to verbal or painful stimulation 44 days after the start of CVVH. The electroencephalogram (EEG) revealed diffuse slowing (5 Hz), with predominant theta and delta waves. Toxic encephalopathy was suspected and plasma ammonia concentration peaked at 148 *μ*g/dL (<90). In parallel, there was a significant increase in LDH, AST, and triglycerides (Figure 1). By contrast, there was no significant change in liver function tests nor evidence for hemolysis.

Among trace elements, plasma copper level was 32 *μ*g/dL (70-140), zinc 83 *μ*g/dL (70-120), iron 23 *μ*g/dL (33-193), and selenium 8.3 *μ*g/dL (5-15).

The plasma acylcarnitine profile revealed an important decrease in free carnitine at 3.2 *μ*mol/L (16-63). Total amount of carnitine was also very low at 7.96 *μ*mol/L (18-84). Intravenous L-carnitine was administered (100 mg/kg over 6 days) (February 2, 2022) and resulted in a rapid improvement of the patient's neurological condition, together with the decrease of ammonia and triglycerides level. Over the following weeks, a significant progress in the weaning from the ventilator was also noted. Free and total plasma carnitine levels were restored at 34.5 and 57.88 *μ*mol/L, respectively; in the ultrafiltrate, the concentration of free carnitine was 31.7 *μ*mol/L. Blood was sampled from the inflow and outflow line of the dialysis circuit, while the patient was receiving a daily supplemental dose of 1 g carnitine. Extraction of plasma carnitine by the circuit was calculated as: [carnitine]_IN_ − [carnitine]_OUT_/[carnitine]_IN_. It corresponded to a plasma extraction of free carnitine of 93% (or 97% when expressed as total carnitine).

## 3. Discussion

Hemofiltration as a mode of CRRT may cause a negative balance in some trace elements and micronutrients, and the effects of prolonged continuous venovenous hemofiltration (CVVH) have been poorly investigated to date [4, 5]. In the studies measuring trace elements during RRT, copper, selenium, and zinc losses were the most frequently reported [6]. In a prospective study on 31 critically ill patients requiring CRRT, clinically effluent losses of copper, iron, selenium, and zinc, as well thiamine and folate, were documented [7]. Our patient had a relatively low plasma copper level, but normal zinc and selenium levels, with the limitation that only a single determination was obtained.

Copper is an essential element required as a cofactor in numerous enzymatic processes. According to some recommendations, the daily dietary intake in an adult should be higher than 2 mg [8]. The effect of copper deficiency on lipid metabolism, including an elevation of plasma lipids (total cholesterol, triglycerides, and phospholipids), has been well documented experimentally [9]. In addition, other complications of copper deficiency include hematologic, neurologic and metabolic disorders, impaired immunity, and heart function.

Carnitine not obtained from food is synthetized endogenously from two essential amino acids, lysine, and methionine. This occurs in the kidney, liver, and brain. Cardiac and skeletal muscle must acquire carnitine from plasma. Carnitine plays a key role in energy metabolism via the transport of long-chain fatty acids from the mitochondrial intermembrane space into the mitochondrial matrix for beta-oxidation in cardiac and skeletal myocytes [10, 11]. Secondary, low plasma carnitine has been observed in critically ill patients with trauma, sepsis, acute organ failure, poor nutritional status, or some drug therapy (e.g., valproate) and has been associated with prolonged ICU stay [12, 13]. Severe muscle atrophy as seen in some ICU patients with prolonged stay could also reduce carnitine stores. In addition, patients receiving long-term feeding with low levels of L-carnitine are also exposed to carnitine deficiency [14, 15]. Carnitine deficiency may be followed by impaired oxidation of fatty acids, impaired immune function, but also altered ventilatory response. The etiology of carnitine deficiency in patients with acute kidney injury (AKI) requiring RRT is likely multifactorial: decreased synthesis due to effluent losses of metabolic precursors (ascorbic acid, amino acids), inadequate dietary intake, or carnitine effluent losses, even if this latter role is still debated [7, 16–18]. Carnitine deficiency may occur with CRRT duration as short as 5-7 days. As shown by this observation, a significant amount of carnitine could be lost in the ultrafiltrate. However, there is no firm recommendation regarding carnitine supplementation in patients receiving CRRT for a prolonged period. In a more chronic setting, a dialysis-related carnitine disorder usually combines anemia, intradialytic hypotension, cardiomyopathy, and skeletal muscle dysfunction [19]. Hyperammoniemia with encephalopathy is also a potential consequence of carnitine deficiency [20, 21]. Indeed, carnitine is also indirectly required for the proper functioning of the urea cycle. When the beta-oxidation is impaired, availability of acetyl-CoA is decreased together with N-acetyl glutamic acid (NAGA), a critical allosteric regulator of carbamoyl phosphate synthetase (CPS-1) activity, resulting in a reduced ammonia clearance. In the present observation, carnitine repletion resulted in a rapid improvement of the patient's neurological condition, together with a decrease in ammonia and triglycerides levels. This would suggest that the role of copper deficiency on impaired fatty acid oxidation was probably modest. Even if there was some discrepancy between CK and AST, LDH levels, muscular injury was a probable complication of acquired carnitine deficiency.

Finally, our patient was initially admitted for a severe COVID-19 pneumonia. The profile of trace elements including zinc, copper, and selenium has been analyzed in COVID-19 patients, but without evidence for a COVID-19-related copper deficiency [22]. Additionally, changes in lipid metabolism have been investigated in COVID-19 patients, but no correlation between carnitine and COVID-19 has been explored [23].

In summary, carnitine deficiency may contribute to sarcopenia and prolonged weaning time from mechanical ventilation in critically ill patients. This case illustrates that the combination of long-term enteral feeding and CRRT may expose the patient to a significant risk of trace elements and micronutrient deficiencies. This could imply a need for closer monitoring and potential supplementation. Awaiting for further prospective and controlled-trials, a possible supplement-level dosing strategy for carnitine would be in the range of 0.5 to 1 g/day for the patients combining long-term tube feeding and CRRT exceeding at least one week.
